# Limited Evidence for Infection of Urban and Peri-urban Nonhuman Primates with Zika and Chikungunya Viruses in Brazil

**DOI:** 10.1128/mSphere.00523-17

**Published:** 2018-01-31

**Authors:** Andres Moreira-Soto, Ianei de Oliveira Carneiro, Carlo Fischer, Marie Feldmann, Beate M. Kümmerer, Nama Santos Silva, Uilton Góes Santos, Breno Frederico de Carvalho Dominguez Souza, Fernanda de Azevedo Liborio, Mônica Mafra Valença-Montenegro, Plautino de Oliveira Laroque, Fernanda Rosa da Fontoura, Alberto Vinicius Dantas Oliveira, Christian Drosten, Xavier de Lamballerie, Carlos Roberto Franke, Jan Felix Drexler

**Affiliations:** a Institute of Virology, University of Bonn Medical Centre, Bonn, Germany; b Charité—Universitätsmedizin Berlin, Corporate Member of Freie Universität Berlin, Humboldt-Universität zu Berlin, and Berlin Institute of Health, Institute of Virology, Berlin, Germany; c Federal University of Bahia, Salvador, Brazil; d Federal University of Reconcavo of Bahia, Cruz das Almas, Brazil; e Wild Animal Triage Center, Salvador, Brazil; f National Center for Research and Conservation of Brazilian Primates, Joao Pessoa, Brazil; g Brasilia Zoo Foundation, Brasilia, Brazil; h Zoobotanic Park Getúlio Vargas, Salvador, Bahia, Brazil; i German Centre for Infection Research (DZIF), Germany; j UMR Emergence des Pathologies Virales (EPV), Aix-Marseille University, IRD 190, Inserm 1207, EHESP, IHU Méditerranée-Infection, Marseille, France; Icahn School of Medicine at Mount Sinai

**Keywords:** alphavirus, chikungunya virus, flavivirus, Zika virus, nonhuman primates

## Abstract

Chikungunya virus (CHIKV) and Zika virus (ZIKV) emerged in the Americas in 2013. Limited antigenic variability of CHIKV and ZIKV may restrict urban transmission cycles due to population protective immunity. In Africa, sylvatic transmission cycles involving nonhuman primates (NHP) are known for CHIKV and ZIKV, causing cyclic reemergence in humans. To evaluate whether sylvatic cycles can be expected in Latin America, we tested 207 NHP collected between 2012 and 2017 in urban and peri-urban settings in Brazil for infection with ZIKV and CHIKV. No animal tested positive for viral RNA in genus-specific and species-specific reverse transcription-PCR (RT-PCR) assays. In contrast, six animals (2.9%) from the families Atelidae, Callitrichidae, and Cebidae showed ZIKV-specific antibodies and 11 (5.3%) showed CHIKV-specific antibodies in plaque reduction neutralization tests (PRNT). Reactivity was monotypic against either ZIKV or CHIKV in all cases, opposing unspecific virucidal activity of sera. PRNT endpoint titers were low at 1:40 in all NHP, and positive specimens did not correspond to the likely dispersal route and time of introduction of both arboviruses. All antibody-positive samples were therefore tested against the NHP-associated yellow fever virus (YFV) and Mayaro virus (MAYV) and against the human-associated dengue virus (DENV) by PRNT. Two ZIKV-positive samples were simultaneously DENV positive and two CHIKV-positive samples were simultaneously MAYV positive, at titers of 1:40 to 1:160. This suggested cross-reactive antibodies against heterologous alphaviruses and flaviviruses in 24% of ZIKV-positive/CHIKV-positive sera. In sum, low seroprevalence, invariably low antibody titers, and the distribution of positive specimens call into question the capability of ZIKV and CHIKV to infect New World NHP and establish sylvatic transmission cycles.

**IMPORTANCE** Since 2013, Zika virus (ZIKV) and chikungunya virus (CHIKV) have infected millions of people in the Americas via urban transmission cycles. Nonhuman primates (NHP) are involved in sylvatic transmission cycles maintaining ZIKV and CHIKV in the Old World. We tested NHP sampled during 2012 to 2017 in urban and peri-urban areas severely affected by ZIKV and CHIKV in Brazil. Seroprevalence and antibody titers were low for both viruses. Additionally, we found evidence for infection by heterologous viruses eliciting cross-reactive antibodies. Our data suggest that urban or peri-urban NHP are not easily infected by ZIKV and CHIKV despite intense local transmission. These data may imply that the ZIKV and CHIKV outbreaks in the Americas cannot be sustained in urban or peri-urban NHP once human population immunity limits urban transmission cycles. Investigation of diverse animals is urgently required to determine the fate of the ZIKV and CHIKV outbreaks in the Americas.

## Abstract (precis)

Since 2013, Zika virus (ZIKV) and chikungunya virus (CHIKV) have infected millions of people in the Americas via urban transmission cycles. Nonhuman primates (NHP) are involved in sylvatic transmission cycles maintaining ZIKV and CHIKV in the Old World. We tested NHP sampled during 2012 to 2017 in urban and peri-urban areas severely affected by ZIKV and CHIKV in Brazil. Seroprevalence and antibody titers were low for both viruses. Additionally, we found evidence for infection by heterologous viruses eliciting cross-reactive antibodies. Our data suggest that urban or peri-urban NHP are not easily infected by ZIKV and CHIKV despite intense local transmission. These data may imply that the ZIKV and CHIKV outbreaks in the Americas cannot be sustained in urban or peri-urban NHP once human population immunity limits urban transmission cycles. Investigation of diverse animals is urgently required to determine the fate of the ZIKV and CHIKV outbreaks in the Americas.

## INTRODUCTION

The Zika virus (ZIKV) is a member of the genus *Flavivirus* in the family *Flaviviridae*, whereas the chikungunya virus (CHIKV) is a member of the genus *Alphavirus* in the family *Togaviridae*. Both arthropod-borne viruses (arboviruses) originated in Africa and were likely introduced into Brazil during 2013, affecting millions of individuals since then (1, 2). The ZIKV circulating in Latin America belongs to the Asian lineage (2). For CHIKV, two separate introductions into Brazil took place. The Asian lineage was introduced in 2013, whereas the eastern, central, and southern African (ECSA) lineage was introduced independently in 2014 (3).

Similarly to the human-associated dengue virus (DENV), the American ZIKV and CHIKV epidemics rely on urban transmission cycles involving predominantly urban *Aedes aegypti* mosquito vectors and humans as vertebrate hosts (4). However, unlike DENV, ZIKV and CHIKV show limited antigenic variability (5, 6). This implies that population immunity following extensive spread in humans may cause the outbreak to stop (7).

In Africa, ZIKV and CHIKV are likely maintained during interepidemic phases in sylvatic transmission cycles involving nonhuman primates (NHP) and forest-associated mosquitoes (8, 9). Which NHP species are involved in these sylvatic transmission cycles is not entirely clear. For ZIKV, seroprevalence of up to 16% hints at frequent exposure of yellow baboons (*Papio cynocephalus*) and African green monkeys (*Chlorocebus pygerythrus*) (10). For CHIKV, virus has been isolated from tantalus monkeys (*Cercopithecus aethiops tantalus*), patas monkeys (*Erythrocebus patas*), Guinea baboons (*Papio papio*) (11), and green monkeys (*Chlorocebus sabaeus*) (12). A high seroprevalence of 72% in these NHP species according to a non-peer-reviewed preprint suggests frequent exposure of these species to CHIKV (13). Blood meal analyses of mosquito vectors competent for ZIKV and CHIKV show preferences for feeding on green monkeys and Guinea baboons (14), which is consistent with evidence for ZIKV or CHIKV infection in these and genetically related NHP species. In Asia, whether and which NHP species are involved in ZIKV and CHIKV transmission cycles is unclear, albeit preliminary evidence hints at infection of different macaque species (15–17).

Similarly, which invertebrate species maintain ZIKV and CHIKV sylvatic transmission cycles in the Old World is not entirely clear. In Africa, potential ZIKV vectors include *Aedes africanus*, *Aedes albopictus*, *Aedes apicoargenteus*, and *Aedes furcifer* (17–19). CHIKV has been detected in numerous mosquito species, including *A. aegypti*, *A. africanus*, *Aedes luteocephalus*, and *Aedes furcifer-taylori* (20). For Asia, the available data on invertebrate hosts sustaining potential sylvatic transmission cycles are scarce.

The ability to extrapolate data from the Old World to potential vertebrate and invertebrate hosts potentially maintaining sylvatic transmission cycles in the Americas is limited. Old World primates (the Catarrhini) comprise the superfamilies Hominoidea, including humans, and Cercopithecoidea (21). Evidence for the ability of ZIKV and CHIKV to infect representatives of both superfamilies may imply a relatively broad host range of these emerging arboviruses within Old World primates. Because New World NHP (the Platyrrhini) arose from Old World ancestors about 36 million years ago (22), susceptibility to ZIKV and CHIKV may be a broadly conserved trait. However, differential susceptibility of New World NHP to yellow fever virus (YFV) illustrates that individual assessments will be required to identify candidate NHP species potentially maintaining ZIKV and CHIKV in the Americas.

On the vector side, *Aedes* species may be among the prime suspects for potential sylvatic transmission cycles. Brazil has approximately 28 *Aedes* species that have only very limited overlap with Old World *Aedes* species other than the widespread *A. aegypti* and *A. albopictus* (23). Indeed, experimental data revealed that *Aedes terrens* mosquitoes, a known vector in American YFV sylvatic transmission cycles, are highly competent for CHIKV (24). However, the YFV vector *Haemagogus leucocelaenus* showed similarly high competence for CHIKV (24), illustrating that potential invertebrate hosts need not be restricted to the genus *Aedes*. Unfortunately, conclusive evidence for vector competence of Neotropical forest-associated mosquito species for ZIKV is scarce to date.

Despite the lack of experimental data, it has been suggested that ZIKV and CHIKV will be able to explore sylvatic cycles in Latin America. The rationale underlying these predictions includes the high number of mosquito and NHP species and their large population sizes in Latin America, as well as the relatively close contact between NHP and humans (25, 26). Another argument that may support an introduction of ZIKV and CHIKV into American NHP is the large die-offs of NHP documented in Brazil during 2016 to 2017, including several species deemed to be robust against YFV, which is a known pathogen of Neotropical NHP (27). Therefore, a report on very high ZIKV detection rates in marmosets (*Callithrix jacchus*) and capuchin monkeys (*Sapajus libidinosus*), available only as a non-peer-reviewed preprint so far, attracted substantial attention in the scientific community and the media (28).

Here, we tested NHP from urban and peri-urban environments showing intense human ZIKV and CHIKV transmission in northeastern and central-western Brazil by molecular and serological methods. Using sera collected before and after the projected introduction of ZIKV and CHIKV into Brazil, we find limited evidence for exposure of Neotropical NHP to these emerging arboviruses.

## RESULTS

**FIG 1:**
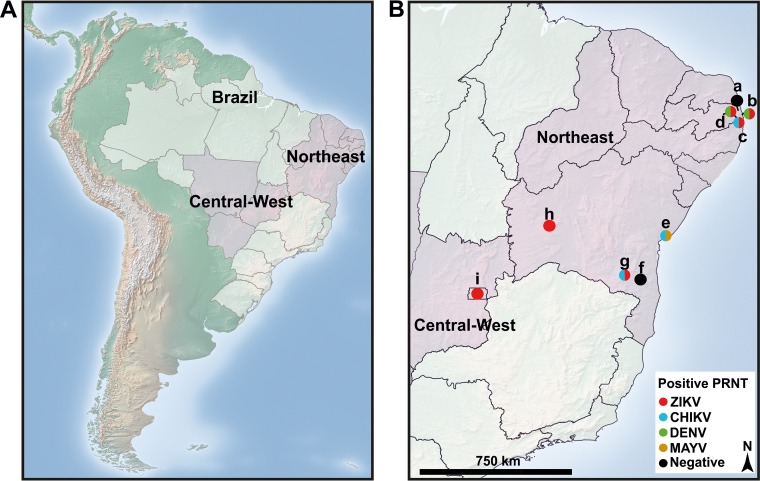
Serological test results across sampling sites. (A) Map of South America with Brazilian regions from which samples originated; (B) locations of Brazilian zoos, national primate centers, and cities in northeastern and central-western Brazilian states where the animals were sampled. Different colors denote positive PRNT results for the viruses under study. Sampling sites are as follows: a, Lucena, Paraíba; b, Cabedelo, Paraíba; c, Santa Rita, Paraíba; d, Sapé, Paraíba; e, Salvador, Bahia; f, Itapetinga, Bahia; g, Vitória da Conquista, Bahia; h, Barreiras, Bahia; i, Brasília, Federal District. The maps were created using QGIS2.14.3 (www.qgis.org). Freely available map data were obtained from www.naturalearthdata.com and http://www.diva-gis.org/gdata.

Samples from 207 NHP were either obtained from local repositories or prospectively sampled in three Brazilian zoos located in the 2.7-million-inhabitant metropolis Salvador and the 2.4-million-inhabitant capital Brasília and from Itapetinga, a northeastern municipality with 75,000 inhabitants. Additional sampling sites were national primate centers in Cabedelo, Salvador, Vitória da Conquista, and Barreiras, as well as urban and peri-urban sites (within 10 km of the city) in northeastern and central-western Brazil, namely, Lucena, Sapé, and Santa Rita, where free-ranging animals were sampled (Table 1; Fig. 1). The samples analyzed in this study represent sampling sites spread across 1,700 km in northeastern and central-western Brazil (Fig. 1). The sampled areas overlap with the areas to which ZIKV and CHIKV were likely introduced upon their arrival in mainland southern America and which showed intense circulation of ZIKV and CHIKV in humans (1, 2). The sample represented 25 NHP species, covering about 20% and all five families of NHP species found in Latin America (Table 1).

**TABLE 1:** Sample characteristics^a^

Family and species^b^ (total no. by family)	No. of samples										Sampling yr (site[s])
	By site:									Total by species	
	a	b	c	d	e	f	g	h	i		
Aotidae (4)											
*Aotus nigriceps*	0	0	0	0	0	0	0	0	4	4	2017 (i)
Atelidae (24)											
*Alouatta caraya*	0	0	0	0	0	0	1	0	6	7	2016 (g)/2017 (i)
*Alouatta belzebul*	1	1	0	1	0	0	0	0	3	6	2013 (d)/2014 (a, b)/2017 (i)
*Alouatta seniculus*	0	0	0	0	0	0	0	0	4	4	2017 (i)
*Ateles marginatus*	0	0	0	0	3	0	0	0	0	3	2013 (e)
*Lagothrix cana*	0	0	0	0	0	0	0	0	2	2	2017 (i)
*Lagothrix* sp.	0	0	0	0	2	0	0	0	0	2	2013 (e)
Callitrichidae (34)											
*Callithrix geoffroyi*	0	0	0	0	0	0	0	0	1	1	2017 (i)
*Callithrix jacchus*	0	0	0	0	9	0	0	0	0	9	2013 (e)/2014 (e)/2016 (e)
*Callithrix kuhlii*	0	0	0	0	0	0	2	2	0	4	2012 (h)/2016 (g)
*Callithrix penicillata*	0	0	0	0	6	0	1	1	3	11	2012 (h)/2013 (e)/2016 (e, g)/2017 (i)
*Callithrix* sp.	0	0	0	0	0	0	1	0	0	1	2016 (g)
*Leontopithecus chrysomelas*	0	0	0	0	1	0	0	0	2	3	2013 (e)/2017 (i)
*Leontopithecus rosalia*	0	0	0	0	0	0	0	0	1	1	2017 (i)
*Mico chrysoleucus*	0	0	0	0	0	0	0	0	1	1	2017 (i)
*Saguinus imperator*	0	0	0	0	0	0	0	0	1	1	2017 (i)
*Saguinus niger*	0	0	0	0	0	0	0	0	2	2	2017 (i)
Cebidae (142)											
*Cebus albifrons*	0	0	0	0	0	0	0	0	1	1	2017 (i)
*Saimiri boliviensis*	0	0	0	0	0	0	0	0	1	1	2017 (i)
*Sapajus flavius*	0	0	21	0	7	0	1	0	0	29	2012 (c, g)/2013 (e)/2014 (c)
*Sapajus libidinosus*	0	0	0	0	1	0	1	2	2	6	2012 (h)/2016 (e, g)/2017 (i)
*Sapajus nigritus*	0	0	0	0	0	0	1	1	0	2	2012 (h)/2016 (g)
*Sapajus robustus*	0	0	0	0	1	0	2	0	0	3	2016 (e, g)
*Sapajus* sp.	0	1	0	0	41	10	21	0	0	73	2012 (a, e, f, g)/2013 (e)/2016 (e, g)
*Sapajus xanthosternos*	0	0	0	0	11	0	16	0	0	27	2012 (g)/2016 (e, g)
Pitheciidae (3)											
*Callicebus cupreus*	0	0	0	0	0	0	0	0	1	1	2017 (i)
*Chiropotes sagulatus*	0	0	0	0	0	0	0	0	1	1	2017 (i)
*Chiropotes satanas*	0	0	0	0	0	0	0	0	1	1	2017 (i)
											
Total	1	2	21	1	82	10	47	6	37	207	

a Sampling sites are designated by letters as defined in the legend to Fig. 1.

b sp., species in cases where morphological typing could not resolve species designation due to frequent hybridization events between different Latin American NHP species (53, 54).

No specimen tested positive for viral RNA in genus-specific nested reverse transcription-PCR (RT-PCR) assays targeting the genera *Flavivirus* and *Alphavirus* (29, 30) or in highly sensitive strain-specific real-time RT-PCR assays for ZIKV and CHIKV (31, 32). This suggested the absence of acute infection with ZIKV and CHIKV or other flaviviruses or alphaviruses in all animals.

In contrast, six animals showed neutralizing antibodies against ZIKV (2.9%). Noticeably, two of these animals were sampled as early as the projected introduction of ZIKV into Brazil in 2013 (Table 2) (33). For CHIKV, we found 11 animals with neutralizing antibodies (5.3%), sampled in 2013 to 2014 and 2016, which again overlaps with the time of the likely introduction of CHIKV into northeastern Brazil (2). Seroprevalence rates for ZIKV and CHIKV did not differ significantly (Fisher’s exact test, *P* > 0.05).

**TABLE 2:** Details of individual samples testing positive^a^

Species	No. of animals positive by PRNT for virus (endpoint titer):					Yr	Total no. of animals sampled per site	Sampling site
	ZIKV	YFV	DENV	CHIKV	MAYV			
*A. belzebul*	1 (1:40)	Neg	1 (1:160)	Neg	NT	2014	1	b
*S. flavius*	1 (1:40)	Neg	Neg	NT	NT	2014	21	c
*S. flavius*	NT	NT	NT	1 (1:40)	Neg	2014	21	
*A. belzebul*	1 (1:40)	Neg	1 (1:40)	Neg	NT	2013	1	d
*Ateles marginatus*	Neg	NT	NT	1 (1:40)	1 (1:40)	2013	3	e
*C. jacchus*	Neg	NT	NT	1 (1:40)	Neg	2013	8	
*Sapajus* sp.	Neg	NT	NT	2 (1:40)	Neg	2013	37	
*S. xanthosternos*	Neg	NT	NT	2 (1:40)	1 (1:40)	2013	11	
*Sapajus* sp.	1 (1:40)	Neg	Neg	Neg	NT	2012	21	g
*S. robustus*	Neg	NT	NT	1 (1:40)	Neg	2016	2	
*Sapajus* sp.	Neg	NT	NT	3 (1:40)	Neg	2016	21	
*Callithrix penicillata*	1 (1:40)	Neg	Neg	Neg	NT	2012	1	h
*A. belzebul*	1 (1:40)	Neg	Neg	Neg	NT	2017	3	i

a Abbreviations and designations: sp., species in cases where morphological typing could not resolve species designation due to frequent hybridization events between different Latin American NHP species (53, 54); Neg, negative; NT, not tested. Sampling sites are designated by letters as defined in the legend to Fig. 1.

Seropositive animals belonged to the Atelidae, Callitrichidae, and Cebidae families, which were also the most abundantly sampled families in this study (Table 1). Seroprevalence for ZIKV and CHIKV in Atelidae was 12.5% (3/24) and 4.1% (1/24); in Cebidae, it was 1.4% (2/142) and 6.3% (6/142), respectively; and in Callitrichidae, it was 2.9% (1/34) for both viruses. No significant difference in the seroprevalence between families was observed for CHIKV (Fisher’s exact test, *P* > 0.05). For ZIKV, only the 12.5% seroprevalence within Atelidae differed significantly from the 1.4% seroprevalence within Cebidae (Fisher’s exact test, *P* = 0.02). Hypothetically, the relatively higher ZIKV seroprevalence in Atelidae may result from differences in the ZIKV-associated mortality between different NHP species, as observed with YFV (34). However, recent studies reported a complete absence of severe disease in different NHP species experimentally infected with ZIKV, albeit the small numbers of infected animals suggest that caution must be used in drawing definite conclusions on ZIKV-associated disease in NHP (35, 36). Alternatively, Atelidae may be more exposed to ZIKV than other NHP families due to differences in feeding preferences of mosquito vectors. However, a sample bias cannot be excluded at this point, even though Atelidae from three different sampling sites spread across >750 km tested positive (Table 2). In sum, whether Atelidae may be more exposed to ZIKV than other NHP families remains to be determined.

**FIG 2:**
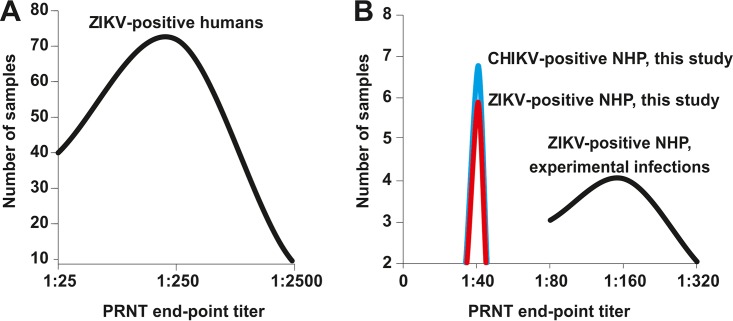
ZIKV-specific and CHIKV-specific PRNT endpoint titers in humans and nonhuman primates. (A) ZIKV-specific PRNT endpoint titers in humans (*n* = 122) as determined previously (37) using the same PRNT protocol as in this study, suggesting comparability of results. One sample with a titer of 1:25,000 is not shown for graphical reasons. (B) ZIKV-specific (red) and CHIKV-specific (cyan) PRNT endpoint titers from this study and from experimental ZIKV infections of New World nonhuman primates (35, 36).

Monotypic reactivity was observed in all 17 sera neutralizing either ZIKV or CHIKV, suggesting that the observed reduction of ZIKV and CHIKV infectivity was not due to potential nonspecific virucidal activity of the analyzed sera. However, plaque reduction neutralization test (PRNT) endpoint titers were invariably low at a 1:40 serum dilution for both ZIKV and CHIKV in all cases. For comparison, PRNT titers in ZIKV-infected humans can be several orders of magnitude higher using the same PRNT protocol (37) (Fig. 2A). Similarly, titers in NHP experimentally infected with ZIKV vary up to 4-fold after seroconversion (Fig. 2B). The low titers that we observed may thus either represent a lower magnitude of ZIKV- and CHIKV-specific antibody responses in New World NHP than in humans or result from cross-reacting antibodies elicited by infection with heterologous flaviviruses or alphaviruses.

Cross-reactivity of antibodies in serological tests is commonly observed within the genus *Flavivirus* (38, 39) and within *Alphavirus* serocomplexes (40) even when using highly specific PRNTs, considered the gold standard for arbovirus serological testing. The most relevant NHP-associated flavivirus in Brazil is YFV, whereas the most relevant human-associated flavivirus in Brazil is DENV. CHIKV belongs to the Semliki Forest serocomplex, which also includes Mayaro virus (MAYV), associated with New World NHP (41). Therefore, we performed PRNTs in all ZIKV-positive samples against YFV and DENV and in the CHIKV-positive samples against MAYV. Two of six ZIKV-seropositive samples (33.3%) neutralized DENV-1 at titers of 1:40 and 1:160 (Table 2). No ZIKV-seropositive samples neutralized YFV. Two of the 11 CHIKV-seropositive samples (18.2%) also neutralized MAYV at low titers of 1:40. Both animals neutralizing MAYV were sampled in the same site in 2013 but belonged to different species, *Sapajus xanthosternos* and *Ateles marginatus*. In sum, the serological reactivity patterns suggested that four of the 17 NHP that tested positive for either ZIKV or CHIKV (23.5%) were exposed to heterologous alphaviruses or flaviviruses.

Finally, ZIKV and CHIKV were likely introduced into different regions of northeastern Brazil upon their arrival in mainland southern America in 2013. ZIKV was introduced to the coastal region of northeastern Brazil, whereas CHIKV was introduced to the hinterland of northeastern Brazil (1, 2). Both ZIKV- and CHIKV-seropositive NHP were found throughout mainland and coastal sampling sites in all sampling years except 2015 (Fig. 1). The geographic distribution of seropositive animals as early as 2013 thus did not match the projected dispersal patterns of CHIKV and ZIKV.

## DISCUSSION

Extensive spread of ZIKV and CHIKV in humans may cause population protective immunity that causes the outbreaks to stop due to lack of acute cases (7). Persistence of ZIKV in the blood of a few individuals for up to 2.5 months has been described (42), and virus may persist in semen for up to 6 months (43). However, reports of prolonged intrahost persistence are scarce, and probably, these isolated cases do not provide a source of virus to allow reemergence on the population level. Cyclic reemergence in humans is possible once a sufficient number of susceptible individuals has become available by birth or migration, such as has been documented for CHIKV in Africa and Asia (44). The time needed for reemergence of urban cycles has been modeled for ZIKV as 10 to 20 years (45) and documented to require several decades for CHIKV in the Old World (46). Sylvatic transmission cycles may maintain ZIKV and CHIKV in the Americas until reemergence in humans is possible.

We found limited evidence for exposure of NHP to ZIKV and CHIKV in urban and peri-urban sampling sites in Brazil. On the one hand, our data may suggest that sylvatic cycles involving Neotropical NHP are possible. Exposure of Brazilian NHP to ZIKV has been suggested by the only preliminary report available to date, which showed a 30% rate of acutely ZIKV-infected, i.e., real-time RT-PCR-positive, animals among peri-urban *Sapajus* and *Callithrix* monkeys (28). The data from this preprint would be consistent with the detection of seropositive animals belonging to the same NHP genera in this study. However, ZIKV seroprevalence in *Sapajus* and *Callithrix* in our study ranged only from 1.4% to 3.8%, which would imply much lower infection rates than those reported in the preliminary study by Favoretto et al. (28) or high mortality of ZIKV-infected NHP, which is not warranted by data from experimental infections (35, 36). Exposure of Neotropical NHP to ZIKV and CHIKV would also be consistent with evidence for infection with heterologous alphaviruses or flaviviruses in only about 24% of seropositive animals. Finally, the low antibody titers that we observed in the field may be in agreement with relatively low 80% PRNT (PRNT_80_) titers observed in experimental infections of squirrel monkeys (*Saimiri boliviensis* and *Saimiri sciureus*), owl monkeys (*Aotus nancymaae*), and marmosets (*Callithrix jacchus*) ranging from 1:80 to 1:320 after seroconversion (35, 36). Our results may thus be consistent with exposure of NHP to ZIKV as hypothesized before (26, 28).

On the other hand, flaviviruses or alphaviruses other than those tested in this study may have elicited cross-reactive antibodies weakly neutralizing ZIKV and CHIKV at a 1:40 serum endpoint dilution, including St. Louis encephalitis virus, West Nile virus, and Eastern and Western equine encephalitis viruses, all of which are known to cause sporadic spillover infections in Neotropical NHP (47). Moreover, ZIKV-specific antibody titers were higher than 1:250 in 50% of tested humans using the same PRNT protocol (37), suggesting comparability of PRNT results between humans and NHP and highlighting the relatively low endpoint titers in NHP. Similarly, the fact that titers were invariably low and restricted to the screening dilution of sera is striking. Normally, PRNT titers should vary on the population level, and they do so in experimental infections and in human cohorts (37). Importantly, the generally low seroprevalence and the absence of an increase in seroprevalence over time are in stark contrast with the intensive ZIKV spread in humans in the same areas, reaching about 60% during 2015 to 2016 in one of the sampling sites of this study (7). Seroprevalence in our study was also lower than that observed in African NHP, at around 16% for ZIKV and 13% for CHIKV (10, 48). Consistently low antibody titers in all seropositive NHP, evidence for possible cross-reactivity in 24% of the ZIKV- and CHIKV-positive sera, and geospatial distribution of positive animals would thus speak against widespread ZIKV or CHIKV infection in Brazilian NHP.

The strengths of our study include the broad sampling of different NHP species, the broad timespan covering the epidemic spread of ZIKV and CHIKV, and the usage of quantitative tools and several heterologous viruses that allowed an evaluation of the specificity of the observed antibody responses. Limitations of our study include the restricted geographical coverage and an irregular number of samples and species tested per site. We thus cannot exclude more frequent exposure of NHP to ZIKV and CHIKV in areas other than those sampled in this study in general and in remote areas in particular, and we cannot exclude that sylvatic transmission cycles are possible in Latin America. That sylvatic transmission cycles are possible in principle is illustrated by the high vector competence of mosquitoes sustaining YFV sylvatic transmission cycles in Neotropical regions for CHIKV (24) and by the susceptibility of different Neotropical NHP to ZIKV during experimental infections (35, 36). Notably, the relevance of NHP for potential ZIKV or CHIKV transmission cycles is highlighted by the lack of significant levels of viremia in numerous nonprimate animals following experimental infections with ZIKV and CHIKV (49, 50).

In sum, we found limited evidence for exposure of urban and peri-urban Neotropical NHP to ZIKV and CHIKV. Our pioneering data may be interpreted as evidence for the exposure of NHP to these emerging arboviruses, which would be consistent with the preliminary data available on this matter (26, 28, 36). However, definite conclusions on the establishment of ZIKV sylvatic transmission cycles are not warranted by our data, given the overall much lower titers of neutralizing antibodies and lower seroprevalence in NHP than in humans (7, 37). Since establishment of sylvatic cycles would change the fate of ZIKV and CHIKV in the Americas, samples that are geographically representative and cover the large genetic diversity of Neotropical NHP are urgently needed. Because sampling of NHP in the wild is restricted for ethical and financial reasons (51), multicenter studies joining groups and animal samples in analogy to prospective cohort studies investigating congenital Zika disease in humans (52) would be highly desirable. If indeed NHP were not involved in sylvatic transmission cycles in Latin America, other wildlife or peri-domestic animals might fill this ecological niche, highlighting the relevance of targeted epidemiological investigations of Neotropical animals for infection with ZIKV and CHIKV.

## MATERIALS AND METHODS

### Sampling and ethical clearance.

Animals were captured with nets and tranquilized using an intramuscular application of ketamine hydrochloride (50 mg/ml, equivalent to 12 to 20 mg/kg of body weight; BioChimico, Germany) and midazolam (5 mg/ml, equivalent to 0.04 to 0.4 mg/kg; Hipolabor, Brazil). After clinical examination, up to 5 ml of blood was collected from the lateral or jugular saphenous veins by a trained veterinarian. Animals were classified according to morphological characteristics such as hair color and size by trained biologists. Sampling and export were approved by Brazilian and German authorities (permit numbers: SISBio/ICMBio, 20610-5/20610-6 and no. 43737-5; Brazilian Ministry of Agriculture, Livestock and Supplies [MAPA], no. 005/2014 and 001/2017; Ethics Committee of the School of Veterinary Medicine of the Federal University of Bahia [CEUA/UFBA], no. 19/2011 and 57/2016; CITES/Brazil, no. 14BR014818/DF and 16BR022343/DF; CITES/Germany, no. E-05004/E00306/17 and E00307/17).

### Laboratory analyses.

All samples were analyzed for viral RNA using broadly reactive nested RT-PCR assays targeting the *Flavivirus* and *Alphavirus* genera (29, 30), as well as highly specific real-time RT-PCR assays for ZIKV (31) and CHIKV (32). Serological assays encompassed plaque reduction neutralization tests (PRNTs) for ZIKV and CHIKV. All sera were heat inactivated (56°C, 30 min) prior to neutralization testing. Briefly, 2 µl of serum was diluted in Dulbecco’s modified Eagle’s medium (DMEM) (containing 1% fetal calf serum) at 1:40 and incubated with 50 plaque-forming units (PFU) of ZIKV (outbreak strain H/PF/2013, Asian lineage, of French Polynesian origin, isolated in 2013) or CHIKV (strain 899, Indian Ocean lineage, of Mauritian origin, isolated in 2006) at 37°C for 1 h. The preincubated virus-serum mixture was used to infect Vero cells in 12-well plates. After incubation for 1 h at 37°C, an agarose-DMEM (containing 2% fetal calf serum) overlay was added. Cells were incubated for 4 days for the ZIKV PRNT and for 2 days for the CHIKV PRNT before formaldehyde fixation and staining. Sera reducing viral PFU by ≥50% were considered positive. Positive sera were further diluted at 1:80, 1:160, 1:320, and 1:640 to determine endpoint titers. Due to the cocirculation of antigenically related arboviruses that could elicit cross-reactive antibodies in infected NHP, ZIKV-positive samples were further tested for antibodies against YFV (using the vaccine strain 17D) and DENV-1 (strain 16007, of Thailand origin, isolated in 1964). CHIKV-positive samples were further tested for antibodies against MAYV (strain TRVL15537, isolated in 1959 in Trinidad and Tobago). Testing for neutralizing antibodies against YFV, DENV, and MAYV was done as described above for the ZIKV PRNT, with the exception that BHK-J cells were used for YFV PRNTs. Incubation times were 3 days for YFV and MAYV and 4 days for DENV.
